# Review of knowledge to guide product development and breeding for sweetpotato frying quality in West Africa

**DOI:** 10.1111/ijfs.14934

**Published:** 2020-12-31

**Authors:** Edward E. Carey, Reuben Ssali, Jan W. Low

**Affiliations:** ^1^ International Potato Center (CIP) c/o CSIR‐CRI Fumesua Kumasi 38785 Ghana; ^2^ International Potato Center (CIP) 00603 Nairobi 25171 Kenya

**Keywords:** Fried product, fries, product profile, sweetpotato, West Africa

## Abstract

This review provides background about sweetpotato in West Africa to identify the current importance and future potential of sweetpotato fried products in the region. We drew on global literature to consider current best practices and health aspects in addition to information from West Africa where frying in the form of large wedges or ‘chunk fries’ is predominant over other forms (i.e. chips (often referred to as crisps in England and the Commonwealth) and ‘French fries’). Chunk fries are produced mostly by female‐run microenterprises selling them as a filling snack to roadside and market customers. Boiling, drying and reconstituting in various foods, pounding and consumption of leaves as a vegetable are also important in the region. Further research will inform the development of a product profile for chunk fried sweetpotato and inform breeding strategies to improve sweetpotato for frying and other uses.

## Graphical abstract

Literature review reveals high potential for commercially important fried sweetpotato in West Africa. Sweetpotato is also widely consumed in other forms. Additional knowledge will inform breeding strategies to improve sweetpotato for frying and other uses.

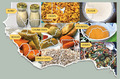

## Introduction

This review of the state of knowledge of fried sweetpotato in West Africa was undertaken to set the stage and underpin the design of subsequent field and laboratory research aimed at developing a product profile to guide breeding for high quality fried sweetpotato in the region (Ssali *et al*., 2021; Dery *et al*., 2021). In West Africa, frying of sweetpotato in the form of large wedges or ‘chunk fries’ is predominant over other forms such as chips (often referred to as crisps in England and the Commonwealth) and ‘French fries’. Frying microenterprises are mainly female‐run, serving chunk fries as a filling snack to customers. The review covers (a) information about production, utilisation and demand concerning fried products; (b) literature on sweetpotato fried product quality, physicochemical properties and processing (including French fries and chips), and health aspects of fried products; and (c) information on the gender and sociocultural context of sweetpotato in West Africa which may contribute to the design of appropriate breeding and product delivery interventions.

## Production of sweetpotato in West Africa

West Africa is home to over 400 million people, with a median age of 18.2 years, and 47.7% dwelling in urban areas (Worldometers, 2020). Agroecologically, it is characterised by parallel zones running east to west, from the northern desert, transitioning to semi‐arid, sub‐humid and humid zones to the south (Dixon *et al*., 2020). Akoroda (2009) reported that considerably more sweetpotato production occurs in the humid savannas (700 to 1000 mm rainfall) than in the forest and more arid savanna zones. West Africa accounts for about 20% of African sweetpotato production (Table 1; FAO, 2018). Sweetpotato in West Africa has largely been an orphan crop from a policy perspective since it does not rank among the top staple food crops except in Sierra Leone and Cape Verde. According to FAO (2018), in Nigeria sweetpotato ranked tenth in terms of both area and production. However, because of its size, Nigeria ranks second globally in area under production and third in production in the widely cited FAOSTAT database. In Ghana, sweetpotato ranked eighteenth in terms of area and twenty‐second in production. However, these statistics for sweetpotato are questionable as they indicate yields of around 2 t ha^−1^ for both Nigeria and Ghana, which are probably too low to be of commercial or even household food security interest to many farmers. In contrast, MoFA‐SRID (2012) reported total production in Ghana was 131 990 t, produced on an area of 9662 ha, with an estimated mean yield of 13.7 t ha^−1^. Akoroda (2003) estimated on‐farm sweetpotato yields at over 15 t ha^−1^ in Nigeria, and authors including Ahmad *et al*. (2014) have reported profitability of sweetpotato farming in Nigeria with the observation that productivity and profitability can be increased through additional use of inputs such as improved varieties and fertiliser. FAO statistics may also overlook production of sweetpotato for primary use as a leafy vegetable, which is important many places in the region, particularly in the Sierra Leone, Guinea and Liberia.

**Table 1:** Sweetpotato production statistics for Africa by region (FAOSTAT, 2018)

Region (no. countries)	yield (t/ha)	Area harvested (×1000 ha)	Production (×1000 t)	Proportion of African production (%)
Central Africa (7)	6.1	402	2447	9.4
East Africa (16)	5.9	1657	9752	37.5
North Africa (3)	22.1	29	640	2.5
Southern Africa (9)	13.7	560	7659	29.5
West Africa (14)	2.8	1952	5503	21.2
Africa	5.7	4600	26 000	28.3^a^
World	11.4	8063	91 945	

^a^ Per cent of global production.

## Utilisation and demand for sweetpotato in West Africa

Most sweetpotato grown in the region is white‐ or yellow‐fleshed, with relatively high dry matter, and varying levels of sweetness. Southern populations tend to prefer less sweet sweetpotato (Akoroda, 2009). Provitamin A‐rich orange‐fleshed sweetpotato (OFSP) is becoming more widespread due to the increasing availability of improved varieties, nutrition awareness, promotion and emerging market opportunities (Low & Thiele, 2020; Adekambi *et al*., 2020a). Adekambi *et al*. (2020b) reported, however, that the taste and low dry matter content of a widely promoted OFSP variety in Ghana were deterrents to its adoption, though these were offset by other desirable attributes.

Sweetpotato in West Africa is predominantly consumed boiled, fried, in foods made from sun‐dried chips or flour, or to a lesser extent, pounded (Akoroda, 2009). In drier areas, at the main harvest at the end of the rainy season, sun‐dried chips are produced, either from raw or parboiled sliced roots (which produces a harder chip resistant to storage pests). Dried chips may be milled into flour (*elubo*, in the Yoruba language of Nigeria), and reconstituted into amala, a thick paste prior to consumption (Fetuga *et al*., 2014). In northern Nigeria, flour from dried chips is used to sweeten *kunun zaki (kunu)*, a popular non‐alcoholic cereal‐based beverage (Gaffa *et al*., 2002). Chunk fries, a filling snack, are a major form of preparation throughout the region. In some urban areas such as Lagos, chips (crisps) are also becoming a popular roadside snack.

Literature on sweetpotato markets in West Africa is very thin, because of the traditionally low priority given to sweetpotato by policy makers, and the informality of markets. There are regions and communities in Nigeria, and Burkina Faso (personal communications, M. Akoroda and K. Some) where sweetpotato production has grown significantly in recent years, serving national and regional markets that have developed organically with limited research support or policy interventions. Further, markets are not static, and studies done a few years ago may not reflect current reality. In Nigeria, production in Kwara State, a traditional sweetpotato‐producing state, has clearly been surpassed by areas in Kano and Kaduna States where access to irrigation for year‐round production has helped transform sweetpotato into a cash crop (Onumah *et al*., 2012; CDA, 2017).

Onumah *et al*. (2012) conducted a value chain assessment of sweetpotato in Nigeria, with focus on Nasarawa, Osun and Kwara states, to identify entry points for research and development investment. They reported that most of the crop was currently sold for urban home consumption where it was mainly boiled or fried. They also recognised a burgeoning microenterprise chunk fry business as well as the potential for higher‐end frozen French fries for the fast‐food sector and chips as a snack. Sweetpotato was considered profitable because of its rapid and high production, and in the case of chunk fries, the low cost of entry into the business. There was a ready market for sweetpotato as it was comparable to yam in terms of production and utilisation, and accessible to the poor when yam was unaffordable. Yellow‐fleshed varieties with high dry matter content predominated due to attractive appearance and low oil consumption during frying, which is related to low moisture or high dry matter content (Lumanlan *et al*., 2020a).

A recent survey of sweetpotato producers, marketers and processors in Nigeria (Kano and Kwara States) and Ghana (Bawku) revealed that much of the sweetpotato crop was sold into urban centres (between 40 and 90% depending on gender of producer and area), and between 58% (in Kano) and 75% (in Kwara) was consumed as fried product (Ssali *et al*., 2021). However, household surveys in sweetpotato‐producing local government areas (LGAs) in Kwara State, Osun and Ogun States in Nigeria showed a heavy reliance on dry chips for flour production, which contributed to household food security and avoided postharvest spoilage of perishable roots (Omoare *et al*., 2015; Adeyonu *et al*., 2016; Alalade *et al*., 2019). Less emphasis was given to frying at the household level since most of the sweetpotato frying was done off‐farm. An unpublished study of 170 marketers and 200 producers in major sweetpotato‐producing LGAs in Kano State (CDA, 2017) revealed the great importance of the crop to both marketers and producers, many of whom reported that sweetpotato marketing or production was their major source of income, with production throughout the year due to availability of irrigation. All 200 producers ranked sweetpotato as their first source of income, with mean sweetpotato production area just under two hectares, but the survey did not report on forms of utilisation.

During a rapid assessment conducted in Nigeria, Ghana and Burkina Faso, Peters (2013) recognised the current and increasing importance of sweetpotato as a commercial crop in these countries and also estimated that a high proportion of sweetpotato use in each country was as chunk fried (Table 2). She made recommendations for commercially and nutritionally oriented interventions, advising that market development and awareness creation of the existing nutritious, but lower dry matter OFSP varieties, should accompany efforts to promote it. The higher dry matter white‐ and yellow‐fleshed varieties could be promoted for their easy market access. For animal feeding, she recommended dual purpose types producing abundant foliage and roots.

**Table 2:** Aspects of sweetpotato (SP) commercialisation and use with implications for variety requirements in Nigeria, Ghana and Burkina Faso (from Peters, 2013; Peters, 2015)

	# yrs. SP as cash crop	Ways SP is consumed	Market access	Variety requirements	Current varieties	Yield (t/ha)
Nigeria	<10	Fried (60%) Boiled (40%)	Four major cities plus local markets	Long shelf life (2–3 weeks) due to slow delivery chain; Sweet taste	2 target national markets	I: Avg: 6.8^a^ II: Avg: 3.7 III: Avg: 3.6
Ghana	5–10	Fried (80%) Boiled (20%)	Spread out around the country	Medium shelf life (1–2 weeks); fry well; Bright colour	Diverse varieties with no champion	North Avg: 7.2 South Avg: 14.1
Burkina Faso	15	Boiled (70%) Fried (30%)	A few particular provinces	High dry matter content, sweet taste	2 target national markets	Avg: 19.5

^a^ These refer to three classes of sweetpotato farmers: Type I specialises in SP production as a cash crop, targets national markets with one or two market‐demanded varieties. Type II produces SP as one of the cash crops, various varieties targeted for local markets. Type III produces SP as a home consumption crop, but still sells a big portion of it.

## Requirements for raw product and preparation methods

Until recently, the importance of chunk fried sweetpotato as an indigenous product seems not to have been appreciated or deemed worthy of study by researchers. In contrast there were numerous publications on both French fries and chips in West Africa, the USA and Asia. In the USA, demand for frozen sweetpotato French fries has created interest in optimising quality both through variety selection and preparation methods. However, methods used by processors of chunk fries in Ghana and Nigeria have recently been described (Ssali *et al*., 2021).

With respect to raw product, soundness of roots is very important given the short shelf life if the root is not cured, and tendency of sweetpotato to become infested with pests and/or to rot. Medium‐ to large‐sized roots with a smooth surface are convenient for peeling and slicing (Ssali *et al*., 2021). Reducing sugars and amino acids in the raw material can contribute to excessive browning during frying, and discoloration of the raw product during preparation can be a problem, due to the presence of polyphenol oxidase (Truong *et al*., 2018).

Reviews of sweetpotato processed products by Padmaja (2009) and Truong *et al*. (2018) cover general practices with respect to both chip and French fry production for formal markets. For chips, slices 0.8 to 2 mm thick are parboiled and partially dried in forced hot air (119 °C) before frying at 143 to 152 °C, draining and packaging. The hot air treatment, leading to partial drying, before frying significantly improves appearance, flavour and texture. For frozen French fries, sweetpotato is sliced into strips roughly 0.9 cm square of varying length and blanched in boiling water containing 1% sodium acid pyrophosphate (SAPP) to prevent oxidative browning. Strips are air‐dried at 120 °C for 5 min (which reduces moisture content, and thus oil consumption, and improves sensory qualities) and then frozen prior to frying. Sometimes, they are par‐fried before freezing. Final frying is done at ~180 °C. In the USA, they are also typically coated with starch‐based materials to improve appearance and exterior crispness for a longer period of time (Truong *et al*., 2018). Standard practices for preparing chunk fries in Ghana and Nigeria are reported by Ssali *et al*. (2021) and do not typically include or drying before frying, though chunks 8 to 11 cm long and 2 to 3 cm thick may be washed in salt solution or lightly salted prior to frying.

## Relating physicochemical to sensory properties and consumer acceptance

Various studies have attempted to relate physicochemical properties of raw and fried sweetpotato to sensory properties and consumer acceptance, particularly to assist breeding programmes to select varieties that make better quality fries, including both French fries and chips. Studies have also reported on new techniques, such as vacuum frying, that significantly reduce oil absorption by the fried products, typically chips. We have summarised details of literature surveyed in Table S1, but present here some selected information related to efforts to understand varietal attributes in relation to chunk fry, French fry, and chip quality.

Walter *et al*. (1997) in the USA evaluated two soft, sweet and three less sweet, firm genotypes for French fry quality. Sensory preference under red lights to mask colour differences showed textural preference for the firm types and taste preference for the soft, sweet types. The authors felt that it would be possible to develop a system to predict fry quality based on texture (shear force) and sugar parameters without the need to resort to use of trained sensory panel. Sato *et al*. (2018) in the USA evaluated French fries made from 16 different sweetpotato genotypes with contrasting characteristics. They found that sensory textural characteristics of French fries were highly correlated with dry matter, alcohol insoluble solids, starch and total sugar in raw sweetpotato, and with instrumental texture measurement (using a French fry rig^1^). They suggested these could be used to help select for improved fry quality in breeding programmes. However, consumer preference attributes to guide the development of a product profile to inform breeding efforts still needed to be described. Recently, Dery *et al*. (2021) related consumer preference to sensory attributes in Ghana and Nigeria, identifying three different consumer segments preferring different varieties based on combinations of appearance, sweetness, flavour and texture. The relationship of physicochemical properties to the consumer preference clusters remains to be reported, but may help to guide selection efforts, when available. It is worth noting that sugar content of sweetpotato can change markedly during cooking due to action of amylases, and that sugar content in cooked sweetpotato is not entirely related to perceived sweetness (Carey *et al*., 2019) so care will need to be taken to verify the predictive value of physicochemical characteristics to fried sweetpotato quality.

Afuape *et al*. (2014) reported on dry matter, starch content and ß‐carotene, and organoleptic traits of sweetpotato chunk fries and boiled roots of fourteen Nigerian breeding selections and released varieties. A semi‐trained panel evaluated degree of liking of colour, mouthfeel, taste, aroma and overall acceptability. Colour and taste contributed significantly to overall liking in fried sweetpotato. The orange‐fleshed, low dry matter Mother’s Delight variety was among the least preferred, but another low dry matter accession, CIP199034.1, gave the most preferred fries, due to aroma and taste. Root flesh colour, followed by taste, contributed significantly to consumer acceptance. Differences in preference among the boiled sweetpotato samples were less marked than among fried samples, though high dry matter sweetpotatoes were generally preferred for both frying and boiling. Sugri *et al*. (2012) used local varieties in Bawku, Ghana to assess consumer preference for boiled sweetpotato and chunk fries in relation to taste, colour, flavour, texture, mouthfeel and overall acceptability. There appeared to be indications of preference clusters for chunk fries, with an OFSP variety preferred due to sweetness and colour by a significant proportion of the population, and less sweet, white‐fleshed varieties preferred by others, similar to the findings recently reported by Dery *et al*. (2021). The authors noted that differences in preference for boiled sweetpotato were masked by frying, and overall acceptance increased in fried samples. Laryea *et al*. (2019) reported on the evaluation of 10 genotypes in Ghana, including released varieties and advanced lines^2^, including oil absorption, carotene loss and sensory evaluation. Fat content of fries varied from 14% to 24%. Three higher dry matter genotypes produced moderately crunchy fries (more yam‐like and considered to be preferred).

Chips are a potentially important emerging product for West Africa. Lv *et al*. (2019) evaluated starch granule size distribution in 21 Chinese sweetpotato genotypes and reported that amylopectin content was higher in roots with a predominance of small (<2.27 µm) and medium‐sized (2.27 to 17.51 µm) starch granules, compared to roots with larger (>17.51 µm) granules. These smaller granules produced both better quality dried slices and fried chips indicating that selection for starch granule size distribution could be a high throughput means to select for superior quality. Ali *et al*. (2012) evaluated chips and French fries of three Caribbean varieties for consumer preference. They reported no difference in preferences among varieties used for chips (which were preferred to French fries). However, there were differences in variety preferences for French fries. The appearance of OFSP was preferred, but taste (perhaps due to lower sugar content) of two white‐fleshed varieties was slightly preferred.

Fetuga *et al*. (2014) looked at optimal time and temperature of frying for a commercially important yellow‐fleshed sweetpotato in Nigeria. Fat content ranged from 19% to 26%, with higher contents giving preferred taste, but conditions were identified (180 °C for 5 min) with lower fat content (22%) but good overall acceptability, important for reducing oil use and extending product shelf life. Nasir *et al*. (2019) looked at optimising OFSP (two varieties: Mother’s Delight and King J) chip production in Nigeria through evaluation of frying times and temperatures. Optimized Mother’s Delight (151 °C for 4.2 min) had lower sensory oiliness and greater consumer acceptance (good appearance, taste and crispness) but also had the highest oil content (30%). In Uganda, Tumuhimbise *et al*. (2013) evaluated the potential for soaking OFSP crisps (chips) (varieties Kakamega and Ejumula) in salt solution before frying to improve shelf life. Carotenoid loss in salted samples was higher than in unsalted samples. Chips fried after 10 min soak in a 2% salt solution were most acceptable and packaged samples could be stored for 2 months at room temperature without quality loss. Gao *et al*. (2014) evaluated attributes of chips made from thirteen sweetpotato genotypes from the USA with diverse textural dry matter, starch properties and starch content. Lower dry matter and lower starch content gave better fracturability measured by texturometer, but sensory analysis was not done. Martin & Rhodes (1984) evaluated raw slices, boiled pieces and chips from 75 genotypes from the Puerto Rico breeding programme and commercial varieties. Using a small trained panel, they rated the appearance and sensory attributes of the raw (1 attribute), boiled pieces (6 attributes) and chips (8 attributes), including liking. Roots that were hard or dry textured after boiling tended to produce chips that were crispy, and moderately sweet boiled roots tended to give good flavour in chips. However, it was not possible to predict acceptable chip quality without frying.

Vacuum frying is a relatively new method, which allows frying at lower temperatures and in the near absence of oxygen. It results in much lower oil uptake by fried products, with better retention of nutrients and flavour, as well as longer life of the cooking oil. In India, Giri *et al*. (2019) reported on process optimisation by response curve methodology and quality attributes of OFSP vacuum fried chips. Vacuum fried chips absorbed ~50% less oil than atmosphere fried chips. Sensory attributes were acceptable for vacuum fried chips. Su *et al*. (2018) reported that combined ultrasound and microwave vacuum frying reduced oil uptake by up to 30%, reduced water activity and shrinkage (extending shelf life and improving product yield) and improved crispness and colour of fried product (4‐mm‐thick chips) compared to vacuum frying. Fan *et al*. (2019) reported osmotic‐ultrasound dehydration pre‐treatment (11.2 min ultrasound, 57% sucrose concentration, 75 min dehydration time prior to microwave vacuum frying at 90°C) gave optimal moisture absorption isotherm and water state. Vacuum frying devices equipped for ultrasound treatment, microwave heating, and centrifugal oil removal are commercially available globally for relatively small batch processing.

## Health considerations of sweetpotato fries

Reducing sugars and asparagine in sweetpotato contribute to browning in fries, and to the production of potentially unhealthy levels of acrylamide. However, blanching with SAPP along with slightly lower fry temperature (165 °C) reduced acrylamide to safe levels (Truong *et al*., 2014).

Oil is essential for the absorption of ß‐carotene by the body, so fried OFSP can be effective for combatting vitamin A deficiency. However, fried foods present a health risk in that they are energy dense and can contribute to obesity, so reduced oil content of fried products is desirable. Lumanlan *et al*. (2020b) recently reported that pre‐drying, a common industry practice, and use of gums, especially xanthan significantly reduced oil absorption in a fabricated chip product, information that may be applied to reduction of oil content in sweetpotato fries. Tumuhimbise *et al*. (2009) compared cooking methods of quartered OFSP roots cut into 2 cm slices, with respect to carotenoid retention and *in vitro* bioaccessibility. They reported significant loss of *all‐trans‐*ß‐carotene by all cooking methods, with an increase in 13‐*cis‐*ß‐carotene. Bioaccessibility was highest in fried slices, more than compensating for loss of total ß‐carotene. Kourouma *et al*. (2019) examined effects of cooking (boil, steam, microwave, roast and fry) on carotenoids and antioxidant activity of OFSP and reported losses of up to 80% ß‐carotene in thinly sliced fried chips, with increases in 9‐*cis‐*ß‐carotene from all cooking methods. Antioxidant activity increased in cooked samples, including fried chips, relative to raw samples. Laryea *et al*. (2029) reported ß‐carotene loss during frying of from 13% to 44% among French fries from three orange‐fleshed genotypes. Giri *et al*. (2019) reported that vacuum fried chips retained roughly 50% more carotenoid than atmosphere fried crisps. Su *et al*. (2018) reported that combined ultrasound and microwave vacuum frying improved retention of anthocyanin in 4‐mm‐thick fried chips compared to vacuum frying.

Oner & Wall (2012) evaluated blanching time and par‐frying of purple‐fleshed sweetpotato to produce oven‐baked or fried final product. The oven‐baked product had 67% less oil and 27% more anthocyanin but was not quite as crispy.

Of potential concern is the glycemic index of sweetpotato which depends on how the roots are prepared. Sweetpotato roots have good levels of dietary fibre (2.5 g/100 g) which promotes good digestion and helps lower blood glucose and blood pressure levels. Roots boiled for 30 min had a low glycemic index of 46, considered very good for health; those roasted can reach a high value of 82. Peeled and fried had a glycemic index around 76, also considered a high value (Bahado‐Singh *et al*., 2006).

## Gender and sociocultural context of sweetpotato in West Africa

References to gender roles in sweetpotato production and utilisation in West Africa are sporadic, providing little guidance with respect to fried sweetpotato, but are reviewed here to provide some context. Women often make decisions about household feeding and so are key actors in variety adoption processes (David, 2015). Sam & Dapaah (2009) reported findings from a baseline survey for the West African Agricultural Productivity Programme (WAAPP) in Ghana. Thirty per cent of respondents were female, probably reflective of male dominance in production, with a lower proportion in the northern part of the country, where women have a harder time entering into agriculture due to cultural and religious constraints. In an adoption/impact study at the end of the WAAPP of sweetpotato varieties released in Ghana, Acheampong *et al*. (2017) reported that, though women were only 16% of those surveyed (only 3% in the northern areas surveyed), they were more enthusiastic adopters of improved varieties, and hence should be intentionally engaged to a greater extent by research and extension programmes.

There are barriers to women engaging in agriculture in northern Ghana (and northern Nigeria), including traditional taboos, and prevailing conservative Islamic strictures. However, women tend to have responsibility for household gardens, and sweetpotato is an interesting crop in this context. Gender‐intentional efforts under the MEDA GROW project in the Upper West Region have successfully encouraged female entrepreneurship^3^, as have efforts under a USAID project called Resilience in Northern Ghana. Onumah *et al*. (2012) reported that frying sweetpotato provided income generation opportunities for a large number of relatively poor women. And Ssali *et al*. (2021) reported that chunk frying was almost exclusively done by women in the areas they surveyed in Ghana and Nigeria.

David & Madu (2014) conducted a situation analysis of gender in sweetpotato production done in three States with contrasting ecologies from north to south in Nigeria. Gender differences were not found in variety preferences. OFSP was largely unknown in the communities studied. Women and men both ranked quality attributes equally in their listing of preferences. Sweetpotato was becoming more commercialised and variety diversity declining as better performing varieties were quickly adopted. Frying was one of the important uses mentioned at each location, and varieties that taste good whether boiled or fried were preferred. Preferred varietal characteristics are summarised by state in Table 3. Other important uses varied by location with incorporation into cassava fufu^4^ (0.25:0.75), dried chips milled into flour for use in amala^5^ or sweetening in kunu. The crop was used for both household consumption and sales (over 50% of harvested production in most cases), though more detailed surveys were recommended.

**Table 3:** Summary of preferred varietal characteristics by male and female farmers interviewed in nine communities where sweetpotato is important in three states in Nigeria (from David & Madu, 2014)

Nasarawa State	Kwara State	Ebonyi State^a^
High root yield Large roots Short maturity period High vine yield White flesh colour Resistance to pests/diseases Marketable Tasty (not too sweet) High dry matter content Low oil absorption when frying (believed to be associated with high dry matter content)	High yielding Early maturing Storable (does not spoil/rot quickly) Not easily attacked by insects or rodents Marketable Sweet (not too sweet) Sticky when pounded Large roots Smooth skin, easy to peel	High yielding Produces many roots Early maturing Grows in any soil Marketable (high price High dry matter (‘solid like yam’) Tasty Multi‐purpose (good for frying and pounding) Smells good when cooked Does not produce gas Quick cooking White flesh colour (so it can pounded with cassava) Does not change colour when cooked Does not soak up oil when fried Not too sweet Good for feeding goats

^a^ Women were more knowledgeable about sweetpotato varieties and their attributes here.

Bidzakin *et al*. (2017) reported on a gender study in six communities in Northern and Upper East Regions (NR and UER) of Ghana by a multi‐disciplinary team under the Gender‐responsive Researchers Equipped for Agricultural Transformation (GREAT) programme. The team interviewed producers, traders and consumers about varietal preferences using quantitative (individual interviews) and qualitative approaches (gender disaggregated focus group discussions). There were striking differences in variety preference by value chain actors between NR (Apomuden, a low dry matter OFSP, preferred) and UER (Obare, a higher dry matter WFSP, preferred). Women and men, however, largely agreed on desired traits within each region. Knowledge of health benefits of OFSP played a large role in variety choice in the NR. Perishability of sweetpotato was a constraint for all value chain actors. The findings did not provide guidance on fry quality.

## Conclusions and Recommendations

This review was conducted to complement detailed studies involving producers, processors and consumers to develop a product profile for chunk fried sweetpotato in West Africa, where fried sweetpotato is increasingly important. The studies, conducted in Nigeria and Ghana used gender disaggregated surveys and consumer preference mapping complemented by descriptive sensory analysis to identify and characterise preferences for chunk fried sweetpotato in Ghana and Nigeria (Ssali *et al*., 2021; Dery *et al*., 2021). Consumer preferences are paramount to the adoption of improved varieties, and chunk fry product profile(s) will help guide ongoing sweetpotato breeding efforts in the region (Thiele *et al*., 2021).

This review drew on global findings, including results from breeding programs, and on economic and socioeconomic information about sweetpotato in West Africa to help inform future breeding and product development efforts. We reviewed reports related to chunk fries, French fries and chips. Noteworthy was the high importance of chunk fried sweetpotato in West Africa, and the general paucity of detailed information about sweetpotato in the region. The finding that multi‐purpose varieties suitable for both frying and other household uses such as boiling and drying were preferred will be very important for breeding strategy consideration, as will the reports of distinct consumer preference clusters for fried products. Depending on size of market segments, this may allow development of distinct product profiles to serve different demographics.

While there were reports relating physicochemical properties of raw and fried sweetpotato to sensory attributes, these were not combined with consumer preference mapping. There is still a need to relate physicochemical characteristics to sensory attributes and consumer preferences in order to improve breeding efficiency. Relationships of dry matter content including starch and cell wall attributes to texture, of constituents such as sugar content and amylase activity to taste, and of carotenoids, anthocyanins to appearance need to be verified for use in high throughput phenotyping in breeding programmes. Future work will need to determine the degree to which these physicochemical characteristics can be used to assist with selection of chunk fries as well as French fries and chips.

The review also highlights that women are heavily involved in the fried sweetpotato value chain in West Africa. Reports of women dominating the processing the processing of chunk fries in Ghana and Nigeria have been attributed to the low capital requirements of the enterprise. Therefore, when developing interventions along the fried sweetpotato value chain the needs of women should receive substantial attention.

Future research and development efforts must also bear in mind that fried product quality can also be improved through appropriate processing steps, such as blanching and drying of roots prior to frying, and using oil at appropriate temperature. These should be considered as part of holistic efforts to improve and deliver healthier and profitable fried sweetpotato options in West African food systems.

## Ethical guidelines

Ethics approval was not required for this research.

## Conflict of interest

The authors declare no conflict of interest in this work.

## Author contribution

**Edward E. Carey:** Conceptualization (lead); Writing‐original draft (lead). **Reuben Ssali:** Writing‐review & editing (equal). **Jan W. Low:** Writing‐review & editing (equal).

### Peer review

The peer review history for this article is available at https://publons.com/publon/10.1111/ijfs.14934.

## Supporting information


**Table S1.** Literature reports on fried sweetpotato with information extracted on product (crisps, French fries or chunk fries), data presented and key findings.

## Data Availability

Literature cited, including unpublished reports, is available upon request from the corresponding author.

## Notes

^1^ French fry rig: a texturometer attachment to measure the force required for breaking multiple fries.

^2^ Fried samples were prepared using the following protocol: No pre‐treatment, frying at 175 °C for 5 min.

^3^ Small investment, big results! https://www.rtb.cgiar.org/news/small‐investment‐big‐results/

^4^ Cassava fufu is a reconstituted product made from elubo flour from fermented cassava pieces after a few days of fermentation.

^5^ Amala is a reconstituted product made from flour from dried yam chips.

## References

[ijfs14934-bib-0001] Acheampong, P.P. , Amengor, N.E. , Nimo‐Wiredu, A. *et al*. (2017). Root and Tuber Crops Technologies Adoption and Impact Study in Ghana: The Case of Improved Sweetpotato Technologies. P. 59 Ghana: Final Report to the West Africa Agricultural Productivity Programme.

[ijfs14934-bib-0002] Adekambi, S.A. , Okello, J.J. , Abidin, P.E. & Carey, E. (2020a). Effect of exposure to biofortified crops on smallholder farm household adoption decisions: The case of orange‐fleshed sweetpotato in Ghana and Nigeria. Scientific African, 8, e00362.

[ijfs14934-bib-0003] Adekambi, S.A. , Okello, J.J. , Rajendran, S. *et al*. (2020b). Effect of varietal attributes on the adoption of an orange‐fleshed sweetpotato variety in Upper East and Northern Ghana. Outlook on Agriculture, 49(4), 311–320. 33239831 10.1177/0030727020950324 PMC7649934

[ijfs14934-bib-0004] Adeyonu, A.G. , Ajala, A.O. , Adigun, G.T. , Ajiboye, B.O. & Gbotosho, O.O. (2016). Determinants of sweet potato value addition among smallholder farming households in Kwara State, Nigeria. Agro‐Science Journal of Tropical Agriculture, Food, Environment and Extension, 15, 17–22.

[ijfs14934-bib-0005] Afuape, S.O. , Nwankwo, I.I.M. , Omodamiro, R.M. , Echendu, T.N.C. & Toure, A. (2014). Studies on some important consumer and processing traits for breeding sweet potato for varied end‐uses. American Journal of Experimental Agriculture, 4, 114–124.

[ijfs14934-bib-0006] Ahmad, I.M. , Makama, S.A. , Kiresur, V.R. & Amina, B.S. (2014). Efficiency of sweet potato farmers in Nigeria: potentials for food security and poverty alleviation. IOSR Journal of Agriculture and Veterinary Science, 7(9), 1–6.

[ijfs14934-bib-0007] Akoroda, M. (2003). Agronomic aspects of root and tuber crops important for estimating production: cassava and sweet potato in relation to time and input variables. Paper 7 In: Proceedings of the Expert Consultation on Root Crop Statistics ‐ Volume II: Invited Papers. http://www.fao.org/3/Y9422E/y9422e00.htm#Contents (accessed 15 July, 2020)

[ijfs14934-bib-0008] Akoroda, M. (2009). Sweetpototato in West Africa. In: The Sweetpotato (edited by G. Loebenstein & G. Thottappilly Springer Netherlands: ), Springer Science+Business Media B.V. 10.1007/978-1-4020-9475-019.

[ijfs14934-bib-0009] Alalade, O.A. , Adisa, R.S. , Olayode, O.O. & Paul, A.B. (2019). Effect of value addition on farm income of sweet potato farmers in Kwara State, Nigeria. Journal of Agricultural Extension, 23, 92–98.

[ijfs14934-bib-0010] Ali, N. , Falade, K.O. & Akingbala, J.O. (2012). Effect of cultivar on quality attributes of sweet potato fries and crisps. Food and Nutrition Sciences, 3, 224–232. Published Online February 2012 (http://www.SciRP.org/journal/fns).

[ijfs14934-bib-0011] Bahado‐Singh, P.S. , Wheatley, A.O. , Ahmad, M.H. , Morrison, E.Y. & Asemota, H.N. (2006). Food processing methods influence the glycaemic indices of some commonly eaten West Indian carbohydrate‐rich foods. British Journal of Nutrition, 96, 476–481. 16925852

[ijfs14934-bib-0012] Bidzakin, J. , Dery, E. , Utoblo, O. , Carey, E. & Low, J. (2017). Gender‐differentiated variety and trait preferences across the SP Value chain: influencing factors and implications for breeding program in Ghana. Unpublished report. CSIR‐SARI and International Potato Center. 46 pp.

[ijfs14934-bib-0013] Carey, E.E. , Swanckaert, J. , Dery, E.K. *et al*. (2019). Developing and deploying non‐ and low‐sweet sweetpotato cultivars for expanding markets. Acta Horticulturae, 1251, 181–188.

[ijfs14934-bib-0014] Center for Dryland Agriculture (CDA) . (2017). Sweetpotato marketer and producer survey, Kano State. Draft report. 48 pp.

[ijfs14934-bib-0015] David, S. (2015). Getting a piece of the pie: an analysis of factors influencing women’s production of sweetpotato in Northern Nigeria. Journal of Gender, Agriculture and Food Security, 1, 1–19.

[ijfs14934-bib-0016] David, S. & Madu, T. (2014). A gender situation analysis of sweetpotato production in Nigeria. Internal report for the Reaching Agents of Change project. HKI and CIP. 59 pp.

[ijfs14934-bib-0017] Dery, E.K. , Carey, E.E. , Ssali, R.T. *et al*. (2021). Sensory characteristics and consumer segmentation of fried sweetpotato for expanded markets in Africa. International Journal of Food Science & Technology. 56(S1), 10.1111/ijfs.14847. PMC7984081 33776243

[ijfs14934-bib-0018] Dixon, J. , Garrity, D.P. , Boffa, J.‐M. *et al*. (2020). Africa through the farming systems lens: context and approach. In: Farming Systems and Food Security in Africa: Priorities for Science and Policy under Global Change (edited by J. Dixon , D.P. Garrity , J.‐M. Boffa , T.O. Williams , T.W.C.A. Amede , R. Lott & G. Mburathi ). pp. 3–37. New York: Routledge.

[ijfs14934-bib-0020] Fan, K. , Zhang, M. & Bhandari, B. (2019). Osmotic‐ultrasound dehydration pretreatement improves moisture adsorption isotherms and water state of microwave‐assisted vacuum fried purple‐fleshed sweet potato slices. Food and Bioproducts Processing, 115, 154–164.

[ijfs14934-bib-0021] Fetuga, G.O. , Ajayi, T.E. & Karim, O.R. (2014). Effect of frying temperature and time on composition and sensory quality of sweet potato crisps. African Journal of Root and Tuber Crops, 11, 17–25.

[ijfs14934-bib-0022] Food and Agriculture Organization of the United Nations . (2018). FAOSTAT statistical database.

[ijfs14934-bib-0023] Gaffa, T. , Jideani, I.A. & Nkama, I. (2002). Traditional production, consumption and storage of Kunu – a non alcoholic cereal beverage. Plant Foods for Human Nutrition, 57, 73–81. 11855622 10.1023/a:1013129307086

[ijfs14934-bib-0024] Gao, M. , Huam, J. , Moallic, C. *et al*. (2014). Structure‐related properties of sweetpotato critically impact the textural quality of fried chips. African Journal of Agricultural Research, 9, 1113–1123.

[ijfs14934-bib-0025] Giri, N. , Pradeepika, C. & Sajeev, M.S. (2019). Process optimization by response surface methodology and quality attributes of orange‐fleshed sweet potato (*Ipomoea batatas* L.) vacuum fried chips. Journal of Food Measurement and Characterization, 13, 22367–2376.

[ijfs14934-bib-0026] Kourouma, V. , Mu, T.‐H. , Zhang, M. & Sun, H.‐N. (2019). Effects of cooking process on carotenoids and antioxidant activity of orange fleshed sweet potato. LWT – Food Science and Technology, 104, 134–141.

[ijfs14934-bib-0027] Laryea, D. , Koomson, D. , Oduro, I. & Carey, E. (2019). Evaluation of 10 genotypes of sweetpotato for fries. Food Science and Nutrition, 2019, 1–10. 10.1002/fsn3.881 PMC6392815 30847138

[ijfs14934-bib-0028] Low, J.W. & Thiele, G. (2020). Understanding innovation: The development and scaling of orange‐fleshed sweetpotato in major African food systems. Agricultural Systems, 179, 102770. 32127727 10.1016/j.agsy.2019.102770 PMC6961970

[ijfs14934-bib-0029] Lumanlan, J.C. , Fernando, W.M.A.D.B. & Jayasena, V. (2020a). Mechanisms of oil uptake during deep frying and applications of predrying and hydrocolloids in reducing fat content of chips. International Journal of Food Science & Technology, 55, 1661–1670.

[ijfs14934-bib-0030] Lumanlan, J.C. , Fernando, W.M.A.D.B. , Karnpanit, W. & Jayasena, V . (2020b). Effects of food gums and pre‐drying on fat content of fabricated fried chips. International Journal of Food Science & Technology, 10.1111/ijfs.14763.

[ijfs14934-bib-0031] Lv, Z. , Yu, K. , Jin, S. *et al*. (2019). Starch granules size distribution of sweet potato and their relationship with quality of dried and fried products. Starch – Stärke, 71, 1800175.

[ijfs14934-bib-0032] Martin, F.W. & Rhodes, A.M. (1984). Sweetpotato variability in boiled slices and fried chips. Journal of Agriculture of the University of Puerto Rico, 68, 223–233.

[ijfs14934-bib-0033] MOFA‐SRID . (2012). Survey report on sweet potato production in Ghana, 2012, 11 pp.

[ijfs14934-bib-0034] Nasir, S. , Olatunde, G. , Adebowale, A. & Aiyelaagbe, I. (2019). Optimization of deep‐fat frying conditions and quality attributes of orange‐fleshed sweetpotato crisps by response surface methodology. Cogent Food & Agriculture, 5, 1658977.

[ijfs14934-bib-0035] Omoare, A.M. , Fakoya, E.O. & Oyediran, W.O. (2015). Value addition of sweet potato (*Ipomoea batatas* L. Lam.): Impending factors on household food security and vitamin A deficiency (VAD) in Southwest and Northcentral Nigeria. IOSR Journal of Agriculture and Veterinary Science, 8, 6–14.

[ijfs14934-bib-0036] Oner, M.E. & Wall, M.M. (2012). Processing conditions for producing French fries from purple‐fleshed sweetpotatoes. Transactions of the ASABE, 55, 2285–2291.

[ijfs14934-bib-0037] Onumah, G. , Dipeolu, A. & Fetuga, G. (2012). Report on sweetpotato value chain study: Exploring opportunities to promote greater exploitation of the benefits of sweetpotato in representative states of Nigeria. Report submitted to the International Potato Center. 71 pp.

[ijfs14934-bib-0038] Padmaja, G. (2009). Uses and nutritional data of sweetpotato. Chapter 11. In: The Sweetpotato (edited by G. Loebenstein & G. Thottappilly Springer Netherlands:), C_Springer Science+Business Media B.V. 10.1007/978-1-4020-9475-011

[ijfs14934-bib-0039] Peters, D. (2013). Assessment Study on Sweetpotato Value Chain and Demand in Nigeria, Ghana, and Burkina Faso. Unpublished report to Report to the Bill and Melinda Gates Foundation. 18 pp. (plus 3 country reports: Nigeria 23 pages, Ghana, 22 pages, Burkina Faso 19 pages).

[ijfs14934-bib-0040] Peters, D. (2015). Sweetpotato value chain development in West Africa: matching products with farmer typology. Chapter 49. In: Potato and Sweetpotato in Africa: Transforming the Value Chains for Food and Nutrition Security (edited by J. Low , M. Nyongesa , S. Quinn & M. Parker ). Pp. 498–507. Boston: CAB International.

[ijfs14934-bib-0041] Sam, J. & Dapaah, H. (2009). West African Agriculture Productivity Programme (WAPP) Ghana. Baseline Survey Report. 159 pp.

[ijfs14934-bib-0042] Sato, A. , Truong, V.‐D. , Johanningsmeier, S.D. , Reynolds, R. , Pecota, K.V. & Yencho, G.C. (2018). Chemical Constituents of Sweetpotato Genotypes in Relation to Textural Characteristics of Processed French Fries. Journal of Food Science, 83, 6073. 10.1111/1750-3841.13978 29178339

[ijfs14934-bib-0043] Ssali, R. , Carey, E. , Imoro, S. *et al*. (2021), Fried sweetpotato user preferences identified in Nigeria and Ghana and implications for trait evaluation. International Journal of Food Science & Technology. 56(S1), 10.1111/ijfs.14847. PMC7984236 33776241

[ijfs14934-bib-0044] Su, Y. , Zhang, M. , Bhandari, B. & Zhang, W. (2018). Enhancement of water removing and the quality of fried purple‐fleshed sweet potato in the vacuum frying by combined power ultrasound and microwave technology. Ultrasonics – Sonochemistry, 44, 368–379. 29680623 10.1016/j.ultsonch.2018.02.049

[ijfs14934-bib-0045] Sugri, I. , Nutsuga, S.K. , Wiredu, A.N. , Johnson, P.N.T. & Aduguba, D. (2012). Kendall’s concordance analysis of sensory descriptors influencing consumer preference for sweet potatoes in Ghana. American Journal of Food Technology, 7, 142–150.

[ijfs14934-bib-0046] Thiele, G. , Dufour, D. , Vernier, P. *et al*. (2021). Review of varietal change in roots, tubers and bananas: consumer preferences and other drivers of adoption and implications for breeding. International Journal of Food Science and Technology; Special Issue: Consumers have their say: assessing preferred quality traits of roots, tubers and cooking bananas, and implications for breeding, 56 (S1), 10.1111/ijfs.14684. PMC7983933 33776222

[ijfs14934-bib-0047] Truong, V.‐D. , Avula, R.Y. , Pecota, K.V. & Yencho, G.C. (2018). Sweetpotato Production, Processing, and Nutritional Quality. Chapter 35 in Handbook of Vegetables and Vegetable Processing, Volume II, Second Edition. Edited by Muhammad Siddiq and Mark A. Uebersax. John Wiley & Sons Ltd.

[ijfs14934-bib-0048] Truong, V.‐D. , Pascua, Y.T. , Reynolds, R. *et al*. (2014). Processing treatments for mitigating acrylamide formation in sweetpotato French fries. Journal of Agricultural and Food Chemistry, 62, 310–316. 24328312 10.1021/jf404290v

[ijfs14934-bib-0049] Tumuhimbise, G.A. , Namutebi, A. & Muyonga, J.H. (2009). Microstructure and *in vitro* Beta Carotene bioaccessibility of heat processed orange fleshed sweet potato. Plant Foods and Human Nutrition, 64, 312–318. 10.1007/s11130-009-0142-z 19908145

[ijfs14934-bib-0050] Tumuhimbise, G.A. , Orishaba, J. , Atukwase, A. & Namutebi, A. (2013). Effect of salt on the sensory and keeping quality of orange fleshed sweetpotato crisps. Food and Nutrition Sciences, 4, 454–460.

[ijfs14934-bib-0051] Walter, W.M. Jr , Collins, W.W. , Troung, V.‐D. & Fine, T.I. (1997). Physical, compositional and sensory properties of French fry‐type products from five sweetpotato selections. Journal of Agricultural and Food Chemistry, 45, 383–388.

[ijfs14934-bib-0052] Worldometers (2020). https://www.worldometers.info/world‐population/western‐africa‐population/ (accessed on June 22, 2020).

