# Supplementary material for: Review of knowledge to guide product development and breeding for sweetpotato frying quality in West Africa
Source: Int J Food Sci Technol. 2020 Dec 31;56(3):1410–8. doi: 10.1111/ijfs.14934 (PMC7986792; doi:10.1111/ijfs.14934)
Supplement: Supplementary file 1 — Table S1. Literature reports on fried sweetpotato with information extracted on product (crisps, French fries or chunk fries), data presented and key findings. [file IJFS-56-1410-s001.docx]

Supplementary Table 1. Literature reports on fried sweetpotato with information extracted on product (**crisps, French fries or chunk fries**), data presented and key findings.

| Reference | Country | Number of genotypes + types^1^ | Product(s), pre-treatment ; frying conditions | Data presented | Key finding |
| --- | --- | --- | --- | --- | --- |
| Afuape *et al.*, 2014 | Nigeria | 14 (OFSP, WFSP, YFSP) | Fried slices (**chunk fries**);  Boiled (methods not detailed) | Proximate (dry matter (DM), starch) Preference for sensory attributes | Lower DM preferred for fry (color and taste contributed) Higher DM preferred for boiling (color, mouthfeel and aroma contributed) Released OFSPs Mother’s Delight (MD) and King J, not preferred for either, though King J ranked better than MD for both. |
| Ali *et al.*, 2012 | UWI, Trinidad | 3 ; 1 OFSP, 2 WFSP | **French fries** (7x1.9x0.64mm) and 1 mm **crisps;** none ; 185°C for 5 min | Various proximate and starch attributes. Preference for sensory attributes | Preference for OFSP French fry appearance, but preference for WFSP taste and texture. No differences in cultivar preferences among crisps. |
| Dery *et al.*, 2021 | Ghana + Nigeria | 5 breeding lines and varieties | **French fries** of varied length. 0.7 x 0.7 cm. Deep fry @180°C for 8 to 10 min, and then drain on paper towel 2 min before wrapping with aluminum foil prior to serving. | Trained sensory panel evaluated French fries using a validated sensory lexicon. Consumer preference assessment in towns in Ghana where sweetpotato is important, and in the capitol city where exposure varies; complemented by trained panel assessment. | Lexicon attributes used by the trained panel: Appearance (4), texture to touch (3), texture in mouth (4), flavor (6), basic tastes (4). Consumer preference mapping showed distinct clusters for OFSP and less sweet W+YSP. Demographic basis of these differences appears to be regional. |
| Euro-Ingredients, 2018 |  |  | **French fry** instruction manual for hand cranked fry cutting machine |  | Recommend blanching whole roots for 3 min, drain and cool. Peel before chipping. Slice using Chip-A-fry. Fries can be kept in covered bucket (no need to add water) until fried at 172 to 190°C. Don’t use same oil for more than 3 batches |
| Fan *et al.,* 2019 | Jiangnan U, Wuxi, Jiangsu, China | PFSP from the supermarket | 4 mm thick **crisps,** given pre-frying ultrasound (40 kHz, 250W, intensity of 14.6 W/l) for 0, 10, 20 or 30 min @25°C and osmotic dehydration for 60, 90 or 120 min @50°C. Osmotic solutions were sucrose at 30, 45 or 60% w/w. Microwave assisted (1000W) vacuum (0.09 MPa) frying @90°C for 15 min in soybean oil for samples from optimized osmotic-ultrasound dehydration treatments. Fried samples centrifuged to remove oil | Response surface methodology was used to evaluate effect of process variables on water loss and solid gain. Moisture adsorption isotherms of pretreated and untreated fried PFSP sliced measured and modeled. Water state of fried slices determined by low field nuclear magnetic resonance (LF-NMR) | Osmotic-ultrasound dehydration pretreatment improved moisture adsorption isotherms and water state of fried product during storage |
| Fetuga *et al.*, 2014 | FUNAAB, Nigeria | 1 YFSP from market | 1.2 mm **crisps**, blanched in 1% NaCl for 2 min, drained and fried; temps from 150 to 180°C for 3-12 min | Moisture, protein, fat, crude fiber, ash, carbohydrate; sensory preference rating of taste, color, flavor, crispness and overall liking | Fat content reduced with temperature and cooking time increase. Overall acceptabililty affected by taste (r=0.93)>flavour (r=0.90)>color (r=0.89)>crispness (r=0.78). Higher fat content gave preferred taste, but 180°C for five min gave good taste, though best was 170°C for 8 min for better overall acceptability and lower fat – important for rancidity and oil use. |
| Gao *et al.*, 2014 | Mississippi, USA | 13 ; (OFSP, WFSP, YFSP) | 1 mm **crisps** ; Control (slices in cold water) + blanching (**crisps** blanched for slightly acidic salty boiling water for 3 min – chilled for 5 min and dried for 4 hours). Cooking at 190°C for 1 min | Texturometer (fracture, puncture and penetration) tests; dry matter, starch and starch properties | Lower dry matter and lower starch contents gave better fracturability. Penetrometer ratings correlated closely with fracturability (30 reps); no sensory evaluation done. |
| Giri et al., 2019 | CTCRI, India | OFSP ST-14 (30% dry matter, carotenoid 9mg/100g) | 1 – 2 mm slices (crinkly **crisps**)**.** Process optimization by response surface methodology using vacuum frying apparatus, and varying frying time, frying temp, and vacuum pressure. | Moisture and oil content, carotenoid content of raw and fried crisps, color and texture (instrumental) | Optimal conditions determined to be 110°C, 16.12 KPa vacuum pressure, and 7 min frying time. Optimize vs atmosphere fried: moisture (9.1 vs 15.4%), oil content (12.3 vs 22.8%), carotenoid (6.8 vs 4.3 mg/100g), color change (20.1 vs 25.0 △E), and texture (1.27 vs 4.37N) |
| Kourouma *et al.,* 2019 | CAAS, Beijing, China | Pushu 32 OFSP from Hebei | SP “cut into pieces” and **boiled, steamed, microwaved** (1150W) for 15, 25, 35, or 45 min. Whole SP **roasted** @190°C fir 15, 25, 35 and 45 min. SP “cut into thin pieces” and **fried** @160°C for 1, 1.5, 2 or 2.5 min. | Proximate composition, carotenoids, vitamin C, antioxidants | Frying reduced ß-carotene content by 80% or more, while other forms of cooking were not as drastic. 9-trans-ß-carotene increased during cooking, with greatest increases in fried product, but did not approach levels of ß-carotene lost. Increased (9Z)- ß-carotene contributed to antioxidant activity. Fat content of fried product increased. The very high loss of ß-carotene might be due to fat solubility. |
| Laryea *et al.*, 2019 | Kumasi, Ghana | 10, including W,Y,P and OFSP Ghana elite lines | **French fries** 0.7x0.7x7.5cm ; Cut, rinsed, patted dry and fried at 175C for 5 min. | Moisture, fat, color, beta carotene of fries; Descriptive sensory for color, crunchiness, hardness, moisture, sogginess, caramel, starch/rawness and oily mouth coat | Fat content of fries varied from 24% (CIP442162) to 14% (CRI-Dadanyuie);TU-Purple, Bohye and CIP440390 produced moderately crunchy fries and had highest score for desired attributes compared to other genotypes (more yam-like). Loss of beta-carotene during frying varied from 13% (Nan) to 44% (Bohye); Apomuden lost 30%. |
| Lv et al., 2019 | Zhejian A&F U., China | 21 genotypes | **Crisps** from washed, peeled, 2 mm slices; boiled in water for 1 min, fried in palm oil 140°C for 4 – 6 min. **Dried sweetpotato** cut into strips 1 x 1 cm and baked @50°C till 15 to 25% moisture. | Starch, amylose and amylopectin content in raw roots, particle size, texture analysis of dried sweetpotato, sensory analysis of crisps (trained panel, but hedonic rating) and product yield | Starch granules divided into small (<2.27µm), medium (2.27 to 17.51 µm) and large size (>17.51 µm). Superior quality of dried sweetpotato and crisps was obtained from genotypes with a higher proportion of small and medium size granules. Small size granules had a greater proportion of amylopectin, while larger starch granules were higher in amylose. |
| Martin and Rhodes, 1984 | Puerto Rico | >310 seedlings screened ; 55 of these selected based on initial screening, and evaluated along with 27 cultivars or breeding lines; | Seedling roots cut and discoloration rated after 15 min. Seedling roots **boiled** 18 min and evaluated.  Selected roots along with cultivars and breeding lines were boiled or fried (1 mm **crisps** were fried in soybean oil @177°C for 4 min.) drained and placed on paper towels | Seedlings classified for oxidation, flesh color, boiled hardness, mouthfeel, sweetness and overall rating and classified into classes based on orange flesh color (not anthocyanin), mouthfeel and sweetness and rating of boiled roots. Selections, cultivars and breeding lines were evaluated independently for various sensory traits and acceptability by a 5-member, small trained panel | 25 of 27 theoretical combinations of color, sweetness and mouthfeel were found in the seedlings. Reasons for rejection of most seedlings were mainly related to discoloration – khaki, gray or non-uniform color. Among the 10 top-rated crisps six were or selections from NCSU, mostly orange, but some few were white. Chips mainly appeal to people on basis of flavor and crispness; seven of top ten were orange, others were cream or white. Authors referred to cookie type chips with rich flavor and potato chips. Though they were not in the top 10, there were genotypes that were good for potato-like chips. |
| Nasir *et al.*, 2019 | FUNAAB, Nigeria | Mother’s Delight (MD) + King J (KJ) | 1-2 mm hand sliced **crisps** ; no pre-treatment ; frying according to central composite design response surface methodology varying time (2.4-6 min) and temp (136-164C) at 5 levels each | Carotenoids, color, sensory quantitative descriptive analysis for color and texture ; and consumer acceptability analyzed | Optimized Mother’s Delight crisps had lower sensory oiliness(?) and higher consumer acceptance. MD optimum at 151.27°C/4.2 min and for KJ at 146.36°C/4.2 min, MD chips had lower moisture content (2.85%), higher oil content (30%), carotenoids, orange-flesh color and crispness |
| Odenigbo et all, 2012 | Quebec, Canada | Five US cultivars | **French fries** ? dimensions not specified but described as discs, fried at 180C for 5 min | *In vitro* starch digestibility, resistant starch, moisture, ash, protein, fat. Predicted glycemic index (pGI) | White Travis and Ginseng Red (OFSP) had higher beneficial starch fractions (resistant starch and slowly digestible starch) and lower pGI even though they had higher total starch. All genotypes were low to moderate pGI |
| Oner and Wall, 2012 | Hawaii, USA | Okinawan PFSP | **French fries** 9.5x9.5x50mm held in water till, blanching for 0, 5 or 10 min in boiling water and cooled in tap water 5 min and drained for 1 min; par frying for 0 or 1 min at 180C; frozen -20°C; fried at 180°C for 3 min or baked at 180°C for 8 min. | Texture analysis (force required to puncture surface), color, moisture, oil, anthocyanins, Semi-trained panel sensory analysis for flavor, texture, appearance and overall quality. | PFSP fries blanched for 10 min, no par-frying and frying had highest sensory preference. Oven baked samples blanched for 10 min, par-fried for 1 min had 65% less oil content and 27% more anthocyanin, and also had good sensory quality (just not as good texture). |
| Padmaja, 2009 | Review |  | Section on **crisps** and **French fries** |  | Crisps : Low dry matter in fresh slices leads to high oil content in fried chips. Partial drying of blanched chips reduces this along with blistering.  French fries: Blanching in 1% SAPP solution, prior to frying and freezing. Crispness improved by 30 min soak of blanched slices in 1% citric acid, surface drying and frying. Low oil content can be achieved by blanching for 3 min in boiling 0.25% SAPP and 0.25% CaCl2 and par-frying at 180°C for 20 sec. |
| Sato *et al.*, 2018 | NC, USA | 16 genotypes with a range of attributes | **French fries.** Whole roots tempered in tap water for 45 min @ 70°C, then cut into 0.9x0.9xcm, blanched for 7 min @75°C, air dried for 10 min@65°C, par-fried for 75 seconds (s) @ 185°C, excess oil removed on paper towel, frozen -20°C till fried for 150s @175°C. | Fresh samples (DM, alcohol insoluble solids (AIS), starch, sugars, amylases); fries (moisture, oil, AIS, starch, total sugar; texture analysis (peak force - puncture, overall hardness – French fry rig), Sensory panel (for attributes in 5 classes) | Variations in sensory texture properties were significantly correlated with chemical components or raw sweetpotato and instrumental texture measurement of French fries. Sensory characteristics (overall hardness, fracturability, outer crispness, inner smoothness, and inner moistness) were highly correlated with DM, AIS, starch and total sugar in raw sweetpotato. The French fry rig was the best texture measurer. Can be used in breeding to predict fry quality. |
| Su *et al.*, 2018 | Jiangnan U., Wuxi, Juiangsu, China | PFSP from market (29.6 % dry matter) | Round crisps 30 mm diameter and 4 mm thickness. Blanched for 3 min @90°C, then rinsed under cold water for 1 min and wiped dry with paper towel. Fried from in increments of 2 min (2 to 16 min in increments of 2 min) to final moisture content of 0.04 kg water/kg solid @90°C using: vacuum frying (VF) @10 KPa; 28 kHz ultrasound (US) @600W combined with VF (USVF); 2450MHz microwave (M) @800W combined with VF (MVF); or combinations of US, M and VF. Chips were centrifuged to remove oil. | Drying kinetics, dielectric properties, moisture state variation using NMR, surface temperature, oil uptake, texture (hardness), shrinkage, color parameters, water activity, total anthocyanins, and scanning electron microscopy of crisps | The combined US-MVF process markedly increased moisture evaporation rate and moisture diffusivity compared to the VF process. Oil uptake was reduced by 16 -34%, water activity and shrinkage were lowered, and texture and color of fried samples improved. Higher US-MVF power level made greater improvement. US600M800VF gave highest anthocyanin levels and retention (80%). SEM revealed more porous and disrupted microstructure in these samples. |
| Sugri *et al.*, 2012 | Bawku, Ghana | Seven local varieties including W and OFSP | Boiled or fried lamellas (**chunk fries**) 3cm x 6 cm | Some root measurements, skin and flesh color; Consumer sensory using hedonic scale for taste, color, flavor, texture, mouth feel, and overall acceptability ; preference ranking and reasons. Kendall’s concordance analysis to independence of variables | Color, aroma, taste and mouthfeel of the OFSP variety Cinkanse-Abiga was liked, while hardness of the WFSP varieties was preferred. Differences among varieties were masked by frying compared to boiling, and overall acceptability of fried SP was higher. Reasons for preference and non-preference appear to indicate preference clusters for sweetness, texture, and differences between fried and boiled. Hardness is a bit ambiguous for fries since the exterior and interior of the root have different preferred textures. |
| Truong *et al.*, 2014 | NC, USA | Covington cured and stored 4 months | **French fries** 0.9 x 0.9 x 9 cm ; Pretreatments: 1. no pretreatment (before frying or par-frying and frying), 2. blanching for 3 min in water @ 95°C, then soaked for 10 min in 0.5% sodium acid pyrophosphate (SAPP), and tap water for 10 min @21°C then air dried for 10 min @65°C; 3. Same as 2, but with 0.4% CaCl2 soak for 10 min @ 62°C before air drying. Strips par-fried for 1 min @ 165°C in canola oil, frozen and then fried for 2,3, or 5 min @165C. | Dry matter, sugars, asparagine, calcium, acrylamide | Pre-treatments reduced acrylamide levels by 10-fold. Untreated fries cooked for 2 min had 125 ng acrylamide/g product (recommended for potato which can range from 60 to 1800 ng/g), but this increased to 452 ng/g after 5 min. 165°C oil temp was recommended to avoid excessive browning. |
| Truong *et al.*, 2018 | Production, processing and nutritional quality | Review | **Crisps** and **French fries**, etc. |  | Crisps : sliced 0.8-2mm., blanched for 2 min at 93°C, drained and partially dehydrated in forced hot air at 119°C. Optimum frying between 143 and 154°C. Vacuum frying at lower temp and de-oiling by centrifuge gives good quality, low fat. Chips then drained and salted.  French fries: Raw slices blanched in boiling 1% SAPP solution, partially dried at 120°C for 5 min, frozen for storage prior to frying. Partial drying reduces oil absorption and improves sensory quality. |
| Tumuhumbise *et al*., 2009 | Uganda | 5 OFSP varieties including Ejumula, Kabode, Vita and Kakamega, plus SPK004/1 | Sweetpotatoes were peeled, quartered and opposite quarters cut into slices 1.5 to 2 cm thick. Slices were **boiled** for 20 min, wrapped in banana leaves and **steamed** for 30 min, **fried** in 170°C sunflower oil for 10 min or **baked** @180°C electric oven. | HPLC analysis of carotenoids, *in vitro* bioaccessibility of ß-carotene, and microstructure analysis | Boiled, deep fried and steamed retained more *all-trans ß*-carotene compared to baked samples for all varieties. Higher levels of 13-*cis-ß*-carotene in processed than raw with levels in proportion to initial *all-trans ß*-carotene levels. *All-trans-ß*-carotene was the predominant carotenoid in all samples. 13-*cis-ß*-carotene was the only trans isomer detected. Deep frying provided greatest bioaccessibility, with over 50% and raw was lowest with ~20%. |
| Tumuhimbise *et al.*, 2013 | Uganda | OFSP- Kakamega and Ejumula | **Crisps** 3.5 mm slices ; soaking for 10 min in 0, 2 or 4% salt solution, drained and fried for 5 min at 180°C. Stored in paper-lined low density polyethylene bags for 3 months in the dark. | Beta-carotene, protein, fat, moisture content; semi-trained sensory panel evaluated color, taste, crispness, mouthfeel and overall acceptability on a hedonic 9 point scale | Carotenoid loss during frying was greater in salt treated samples. Crisps treated with 2% salt were most acceptable. Freshly made Ejumula was better than Kakamega, but they were equal after two months of storage, with acceptability of Kakamega having increased. After three months, quality was reducing, but 2% salt-treated crisps were still rated highest. Non-salt-treated had highest storage stability. |
| Ukpabi *et al.*, 2012 | Nigeria | 4 OFSP and 1 yellow introduced, but clear misidentifications since some known WFSP were listed as OFSP | **Crisps** sliced mechanically at 1 to 2 mm, salted (no description) and deep fried at 140 to 150°C for 2 to 3 min. | Dry matter content, beta- carotene of raw and fired chips, peel loss. 20 semi-trained consumer panelists used a 7-point hedonic scale to rate for crispness, sugariness, and overall acceptability | The most acceptable crisp scored 4.3 on a 1 to 7 scale. It was also preferred for sugariness and crispness. Discoloration of crisps was a problem with one genotype. Dry matter content ranged from 21 to 38% with the best genotype reported to have 36% DM. |
| Walter *et al.*, 1997 | NCSU, USA | 2 sweet, soft  3 less sweet, firm | **French fries** : 0.9 x 0.9 x variable length from cured, stored roots. Blanched in boiling 1% SAPP, air drying and freezing prior to frying. Frying at 180°C for 3 min (oil temp dropped to 150 and ended at 155°C) ; Fries drained for 30s and put in paper-lined containers covered with aluminum foil and held at 60°C until evaluated within 15 min. Shear force measured on room temp samples. | DM, sugar, AIS, % intercellular space (SG) by vacuum method, cell size by microscopy, pectic substances, oil content, shear force, Sensory texture-flavor profile trained panel (4 taste notes, 7 texture notes); preference panel (under red light) for flavor, texture and overall | Texture of the firm ones was preferred, while flavor of the sweet-soft selections. Looked at correlations and found some, but further work needed to develop a system for prediction of fry quality based on instrumental analysis. |

^1^Sweetpotato flesh color: Orange fleshed sweetpotato (OFSP), yellow fleshed sweetpotato (YFSP), white fleshed sweetpotato (WFSP), purple fleshed sweetpotato (PFSP)
